# Structural and Rheological Properties of Nonedible Vegetable Oil-Based Resin

**DOI:** 10.3390/polym13152490

**Published:** 2021-07-28

**Authors:** Nurul Huda Mudri, Luqman Chuah Abdullah, Min Min Aung, Dayang Radiah Awang Biak, Rida Tajau

**Affiliations:** 1 Department of Chemical and Environmental Engineering, Faculty of Engineering, Universiti Putra Malaysia, Serdang 43400, Selangor, Malaysia; dradiah@upm.edu.my; 2 Radiation Processing Technology Division, Malaysian Nuclear Agency, Kajang 43000, Selangor, Malaysia; rida@nm.gov.my; 3 Institute of Tropical Forestry and Forest Products (INTROP), Universiti Putra Malaysia, Serdang 43400, Selangor, Malaysia; minmin_aung@upm.edu.my; 4 Centre of Foundation Studies for Agricultural Science, Universiti Putra Malaysia, Serdang 43400, Selangor, Malaysia; 5 Institute of Advanced Technology, Universiti Putra Malaysia, Serdang 43000, Selangor, Malaysia

**Keywords:** jatropha oil, proton NMR, rheology, encapsulation, master curve graph

## Abstract

Jatropha oil-based polyol (JOL) was prepared from crude Jatropha oil via an epoxidation and hydroxylation reaction. During the isocyanation step, two different types of diisocyanates; 2,4-toluene diisocyanate (2,4-TDI) and isophorone diisocyanate (IPDI), were introduced to produce Jatropha oil-based polyurethane acrylates (JPUA). The products were named JPUA-TDI and JPUA-IPDI, respectively. The success of the stepwise reactions of the resins was confirmed using ^1^H nuclear magnetic resonance (NMR) spectroscopy to support the Fourier-transform infrared (FTIR) spectroscopy analysis that was reported in the previous study. For JPUA-TDI, the presence of a signal at 7.94 ppm evidenced the possible side reactions between urethane linkages with secondary amine that resulted in an aryl-urea group (Ar-NH-COO-). Meanwhile, the peak of 2.89 ppm was assigned to the α-position of methylene to the carbamate (-CH_2_NHCOO) group in the JPUA-IPDI. From the rheological study, JO and JPUA-IPDI in pure form were classified as Newtonian fluids, while JPUA-TDI showed non-Newtonian behaviour with pseudoplastic or shear thinning behaviour at room temperature. At elevated temperatures, the JO, JPUA-IPDI mixture and JPUA-TDI mixture exhibited reductions in viscosity and shear stress as the shear rate increased. The JO and JPUA-IPDI mixture maintained Newtonian fluid behaviour at all temperature ranges. Meanwhile, the JPUA-TDI mixture showed shear thickening at 25 °C and shear thinning at 40 °C, 60 °C and 80 °C. The master curve graph based on the shear rate for the JO, JPUA-TDI mixture and JPUA-IPDI mixture at 25 °C, 40 °C, 60 °C and 80 °C was developed as a fluid behaviour reference for future storage and processing conditions during the encapsulation process. The encapsulation process can be conducted to fabricate a self-healing coating based on a microcapsule triggered either by air or ultra-violet (UV) radiation.

## 1. Introduction

Polyurethane acrylate (PUA) is a subclass of polyurethane (PU) where the polyol and diisocyanate have been reacted and end-capped with an acrylate functional group during the termination reaction [1]. The presence of the acrylate functional group allows the utilisation of PUA in a UV-curing technique in coating production. This technique offers instant drying, high curing rate, broad formulating range, low-energy consumption and less capital requirement to install the curing equipment compared to the conventional thermal method [2]. The PUA itself is now rapidly being researched as the new generations were developed such as hybrid PUA [3], hyperbranched PUA [4,5], waterborne PUA [6] and self-healing PUA [7] to suit the specific coating applications.

In the coating industry, the utilisation of vegetable oil as a precursor and binder has begun over the last few decades. This is because vegetable oils are cost-efficient and environmentally friendly compared to petroleum-based chemicals. This solves the issues related to petrochemical resin such as price fluctuation and the release of hazardous volatile organic compounds (VOCs) into the environment [8]. Vegetable oils have a unique property where the triglyceride component includes an unsaturated component that permits chemical modification such as epoxidation, hydroxylation, isocyanation, acrylation and transesterification [9]. The triglycerides consist of glycerol and a variety of fatty acids which are different based on the types of oil and their locality [10]. A few vegetable oils have been studied to produce natural-based polyol in manufacturing various types of PU such as soy [11], palm [12], olive, canola, castor [13] and sesame [14]. However, the use of these edible oils has faced criticism regarding the utilisation of food supplies for nonfood applications.

Jatropha oil (JO) has emerged as one of the most suitable candidates for producing polyol for PUA applications as it is classified as a nonedible oil. It contains a phorbic ester compound that is poisonous for oral consumption [15,16]. JO is extracted from the seed of the fruit of *Jatropha curcas*, which has currently has been widely planted in Latin America, Africa, India and South East Asia [17]. It contains about 79% of unsaturated fatty acid, which is mainly contributed by oleic acid (43.5%) and linoleic acid (35.2%) [18]. Apart from that, it has an iodine value (IV) of around 94–120 mg/g [10,18], which reflects its sensitivity during chemical modification. The IV of JO is slightly lower than that of soy oil (120 to 143 mg/g) [19] but higher than those of palm oil (44 to 58 mg/g) [20] and olive oil (79 to 88 mg/g) [19].

In the previous study, we reported the synthesis of jatropha oil-based polyol (JOL) via the epoxidation and hydroxylation route. JOL was then reacted with two types of diisocyanate known as 2,4-toluene diisocyanate (2,4-TDI) and isophorone diisocyanate (IPDI). As a result, two series of JPUA were produced and identified as JPUA-TDI and JPUA-IPDI, respectively. The mechanical properties for both the UV-curable JPUA-TDI and JPUA-IPDI-based films such as hardness, contact angle, haze and transmittance was discussed. The ratio of 35:65 (wt.%) between the monomer and JPUA was determined as the best ratio based on the performance of the mechanical properties [21].

For fabrication of a self-healing coating, the pure JO, and the selected uncured coating formulations of JPUA-TDI and JPUA-IPDI were used as a healing agent or core content of the microcapsule in the next stage of the experiment. The microcapsules act as a reservoir that contains an active healing agent and is distributed evenly in the coating material. As the coating is exposed to scratches and cracks, the microcapsules will rupture and release the active agent into the crack volume. The active agent will react with the catalyst or natural condition such as atmospheric moisture, oxygen and sunlight to activate the polymerisation reaction in order to repair the crack surfaces [22].

Several studies have been conducted on other types of oil such as linseed [23,24], tung [25,26], palm [27,28] and soy [29] for the microcapsule-based self-repairing mechanism. To the best of the knowledge of the authors, no study has been conducted related to JO-based resin for self-healing coating application using the encapsulation technique. This procedure was conducted via the oil-in-water emulsification process [22]. Based on previous studies, experimental parameters such as agitation rate and temperature have been demonstrated to affect the morphology, core content and size distribution of the microcapsules [30,31,32]. In addition, several studies have also reported on the agglomeration problem during the encapsulation process [33,34]. As stated by Gan and Shahabudin, the viscosity of the selected healing agent must be taken into consideration during the encapsulation process [35].

As a solution, a rheology study was proposed to predict the fluid behaviour of JO and the selected uncured formulation mixture of JPUA-TDI and JPUA-IPDI in designing the microcapsule. Currently, no rheology study has been conducted to understand the fluid behaviour of the healing agent before the encapsulation process is carried out. The rheological properties provide information regarding fluid behaviour such as viscosity and shear stress at different temperatures and shear rates. This will also involve the correlation between the rheological properties with the chemical structure [36]. In addition, the establishment of a common master curve based on either viscosity or shear rate, which is measured at different temperatures, was reported to create practical information regarding fluid behaviour for future processing conditions [37].

In this paper, the aim was to further investigate the chemical structure of JO-based resin during the synthesis reaction of epoxidation, hydroxylation and isocyanation via ^1^H NMR analysis. Next, the chemical structure of the pure JO and uncured formulation mixture of JPUA-TDI and JPUA-IPDI were studied by relating them to the rheological properties and experimental parameters such as the variation in shear rate and temperature. Finally, the shear rate master curve graph for JO and both formulation resins of the JPUA-TDI mixture and JPUA-IPDI mixture was developed.

## 2. Materials and Methods

### 2.1. Materials

Crude Jatropha oil was supplied by Biofuel Bionas Malaysia Sdn. Bhd., Kuala Lumpur, Malaysia. The chemical composition of the jatropha oil is listed in Table 1. Benzophenone (IUPAC name: diphenylmethanone) was purchased from Acros Organic (Geel, Belgium). Formic acid (98%), hydrogen peroxide (30%), methanol (99.8%) and sulphuric acid (95%) were shipped by R&M Chemicals, Chandigarh, India. 2,4-Toluene diisocyanate (IUPAC name: 2,4-diisocyanato-1-methylbenzene) (2,4-TDI) (95%), isophorone diisocyanate (IUPAC name: 5-isocyanato-1-(isocyanatomethyl)-1,3,3-trimethylcyclohexane) (IPDI) (98%), dibutyltin dilaurate [IUPAC name: (dibutyl(dodecanoyloxy)stannyl) dodecanoate] (DBTDL) (95%), hydroxyethyl methacrylate (IUPAC name: 2-hydroxyethyl 2-methylprop-2-enoate) (HEMA) and trimethylolpropane triacrylate (TMPTA) were provided by Sigma-Aldrich, Darmstadt, Germany. All chemicals were used as received.

### 2.2. Synthesis of Jatropha Oil-Based Polyol (JOL)

Details of the synthesis procedure of JOL and JPUA have been reported in the previous study of the same authors [21]. In brief, the preparation of JOL from crude Jatropha oil (JO) was conducted via epoxidation and a hydroxylation step. The crude JO consisted of an unsaturated carbon double bond which was reported to appear at the absorption band of 3012 cm^−1^ under FTIR analysis.

During the epoxidation step, 1 mol of JO was mixed with 0.6 mol of formic acid at 40 °C with a stirring rate of 300 rpm for 15 min. Then, 1.7 mol of hydrogen peroxide was added in a dropwise manner and the temperature was increased gradually to 60 °C. The mixture was allowed to complete the reaction over a total duration of 4.5 h. The mixture of epoxidised jatropha oil (EJO) was cooled and moved into a separating funnel. The unwanted aqueous layer was then carefully discarded. The remaining EJO was washed with distilled water until it was free from acid. The excess water was removed using a rotary evaporator with a temperature of 60 °C. The disappearance of the peak at 3012 cm^−1^ and the appearance of a new peak at 824 cm^−1^ of the FTIR spectra were reported. This evidenced that the carbon double bond (C=C) had been converted to the epoxy group (C–O–C) to produce epoxidised Jatropha oil (EJO) [21].

For the hydroxylation step, EJO was reacted with methanol in the presence of an acid catalyst. Initially, 9 mol of methanol was mixed with 1 mol of 0.07% of sulphuric acid at 40 °C and 300 rpm for 15 min. Next, 10 mol of EJO was added into the solution and the reaction was mixed at 65 °C and 300 rpm for 30 min. The mixture was cooled and then transferred into a separating funnel to remove the aqueous layer. The JOL was washed with distilled water until it reached neutral pH. To purify the JOL, the solution was run under a vacuum distillator at 60 °C to remove the excess water and methanol. For FTIR analysis, the broad peak of the hydroxyl (OH) group appeared at 3435 cm^−1^, which indicated that EJO was converted into Jatropha oil-based polyol (JOL) [21].

### 2.3. Synthesis of Jatropha Oil Polyurethane Acrylate (JPUA)

To produce JPUA, JOL was reacted with two types of diisocyanates, namely 2,4-TDI and IPDI via an isocyanation reaction. The reaction was performed based on a stoichiometric calculation of (1:1:1) for JOL:IPDI:HEMA, respectively. As isocyanates are sensitive to moisture, the reaction was conducted in a four-neck flask in a nitrogen-purged system.

Next, 1% (*w*/*w*) of DBTDL was mixed with JOL at 350 rpm and 60 °C for 15 min. Then, IPDI was added slowly via a dropping funnel to start the isocyanation reaction. Upon completion of the dropping of IPDI, the mixture was stirred continuously for another 2 h. From FTIR monitoring, the isocyanate peak (N=C=O) at 2270 cm^−1^ was observed during the initial addition of IPDI. The disappearance of the peak at 2270 cm^−1^ after 2 h indicated that all isocyanates had been consumed. Moreover, the peak of (–OH) at 3435 cm^−1^ was shifted to 3350 cm^−1^, which corresponds to (–NH) amide bending. New peaks of (–NH) amide stretching and carbonyl (C=O) were observed at 1532 at 1720 cm^−1^, respectively [21].

The reaction was terminated via an acrylation reaction with HEMA. Before HEMA was added, the temperature was set to 40 °C and HEMA was introduced into the mixture in a dropwise manner. Then, the temperature was raised up to 70 °C and the reaction was continued for 1 h to complete the reaction of JPUA-IPDI. The peak of the acrylate double bond (–CH=CH_2_) was observed at 1663 cm^−1^ and the acrylate vinyl functionality of (CH_2_=CH–COO–) appeared at 815 cm^−1^ under FTIR analysis, which proved that the acrylation reaction had occurred [21]. 

The same procedure was repeated for 2,4-TDI-based isocyanates to produce JPUA-TDI with the same stoichiometric ratio. However, no DBTDL catalyst was added, as 2,4-TDI is already highly reactive.

### 2.4. ^1^H Nuclear Magnetic Resonance (NMR) Spectroscopy

The chemical structures of JO, EJO, JOL, JPUA-TDI and JPUA-IPDI were predicted using ^1^H NMR analysis (Bruker Avance III 600 MHz, Rheinstetten, Germany). DMSO solvent was used to dilute the sample and also as an internal standard during the analysis.

For synthesis of the polyol, the presence of the functional groups such as the carbon double bond, epoxy and hydroxyl groups during the stepwise reactions was determined as the function to one mole of triglyceride in JO, EJO and JOL, respectively. The percentages of the functional groups were calculated based on the integration of the peak areas of the ^1^H NMR spectrum, as shown in Equation (1) [38].

(1) $$\mathrm{Functional}\;\mathrm{group}\;(\%)=\frac{\mathrm{Af}}{\mathrm{Atotal}}\times 100\%$$

where A_f_ is integration area of the functional group of either the carbon double bond, epoxy or hydroxyl group, and A_total_ is the total integration area of the triglyceride.

### 2.5. Preparation of JO, JPUA-TDI, JPUA-IPDI and Their Mixtures

The rheological properties of the JO, JPUA-TDI and JPUA-IPDI were compared between their original form and the formulation mixture. The components of each sample were prepared as listed in Table 2. For the JPUA-TDI mixture and JPUA-IPDI mixture, TMPTA and benzophenone were used as a reactive diluent and photoinitiator, respectively. From our previous study, the ratios of 65 (wt.%) of JPUA-TDI or JPUA-IPDI and 35 (wt.%) of TMPTA were selected as the best ratio formulation based on their performance in terms of the mechanical properties [21]. In the mixture, 4% (*w/w*) of benzophenone was added based on the total amount of either JPUA-TDI or JPUA-IPDI and TMPTA. The benzophenone produced a radical species only when it was exposed to UV light to start the radical polymerisation reaction. Therefore, all samples were put in amber bottles to avoid exposure to UV sunlight. All components were mixed using a mechanical stirrer at 300 rpm for 15 min at room temperature until it reached a homogenous condition.

### 2.6. Rheological Properties

The rheological properties of the resins were determined using a Haake Mars Rheometer (Thermo Scientific, Waltham, MA, USA). Then, 1 g of resin was laid on a flat sample platform for a plate–plate geometry mode. The gap between the spindle and platform was set at 1 mm. The spindle (PP60) was then rotated on the sample. First, the viscosity and shear stress of pure JO, JPUA-TDI and JPUA-IPDI were measured at 25 °C with the shear rate range set between 0 and 100 s^−1^. 

Viscosity measures the liquid flow resistance and is defined as force (F) divided by area (A); (F/A). The speed of a moving liquid or (dv/dr) is known as the shear rate, whilst shear stress is the force that acts parallel to the cross-section of the material. The correlation between viscosity (η), shear rate (γ) and shear stress (σ) is expressed in Equation (2) below [39]:

(2) $$\eta =\frac{\sigma }{˙\gamma }$$

The viscosity and shear stress of the JO, JPUA-TDI mixture and JPUA-IPDI mixture were compared at four different temperatures, namely 25 °C, 40 °C, 60 °C and 80 °C. The shear rate values were set in the range from 0 to 100 s^−1^ for all samples. For a greater understanding of the relationship between shear stress in the range of tested shear rates, the power law equation was used. The equation is expressed as Equation (3) below.

σ = k ˙γ^n^ (3)

where σ is the shear stress (Pa); k is the consistency index; ˙γ is the shear rate (s^−1^); and n is the flow behaviour index.

Based on the calculation, the consistency index (k) and flow behaviour index (n) for JO, the JPUA-TDI mixture and JPUA-IPDI mixture were determined from the graph of ln shear stress versus ln shear rate.

Next, the master curve of shear stress–shear rate was constructed with the aim to simultaneously fit the data of different temperatures and to predict the flow behaviour. It offers accurate and practical results rather than fitting the data separately [37]. Based on the k and n value, the calculated shear rate was expressed based on Equation (4) [40].

(4) $$˙\gamma =\left(\frac{\sigma }{K}\right)1/n$$

The shift factor, aT, was calculated based on the calculated shear rate divided by the reference shear rate (at 60 °C). The compilations of the relationship between the shear stress and shear strain of JO, the JPUA-TDI mixture and JPUA-IPDI mixture at 25 °C, 40 °C, 60 °C and 80 °C were analysed.

## 3. Results and Discussion

### 3.1. ^1^H NMR Analysis

**Figure 1:**
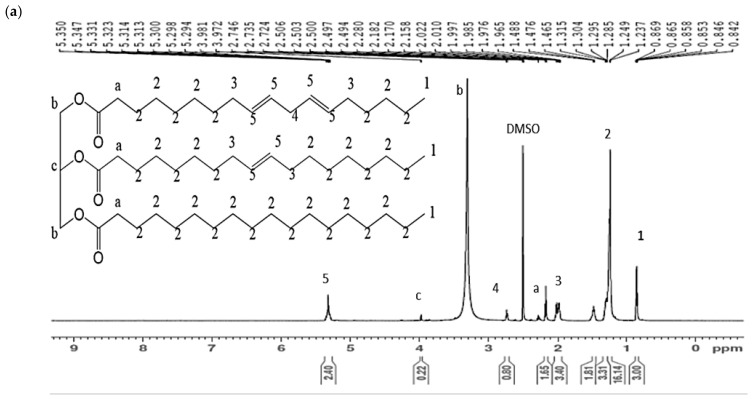

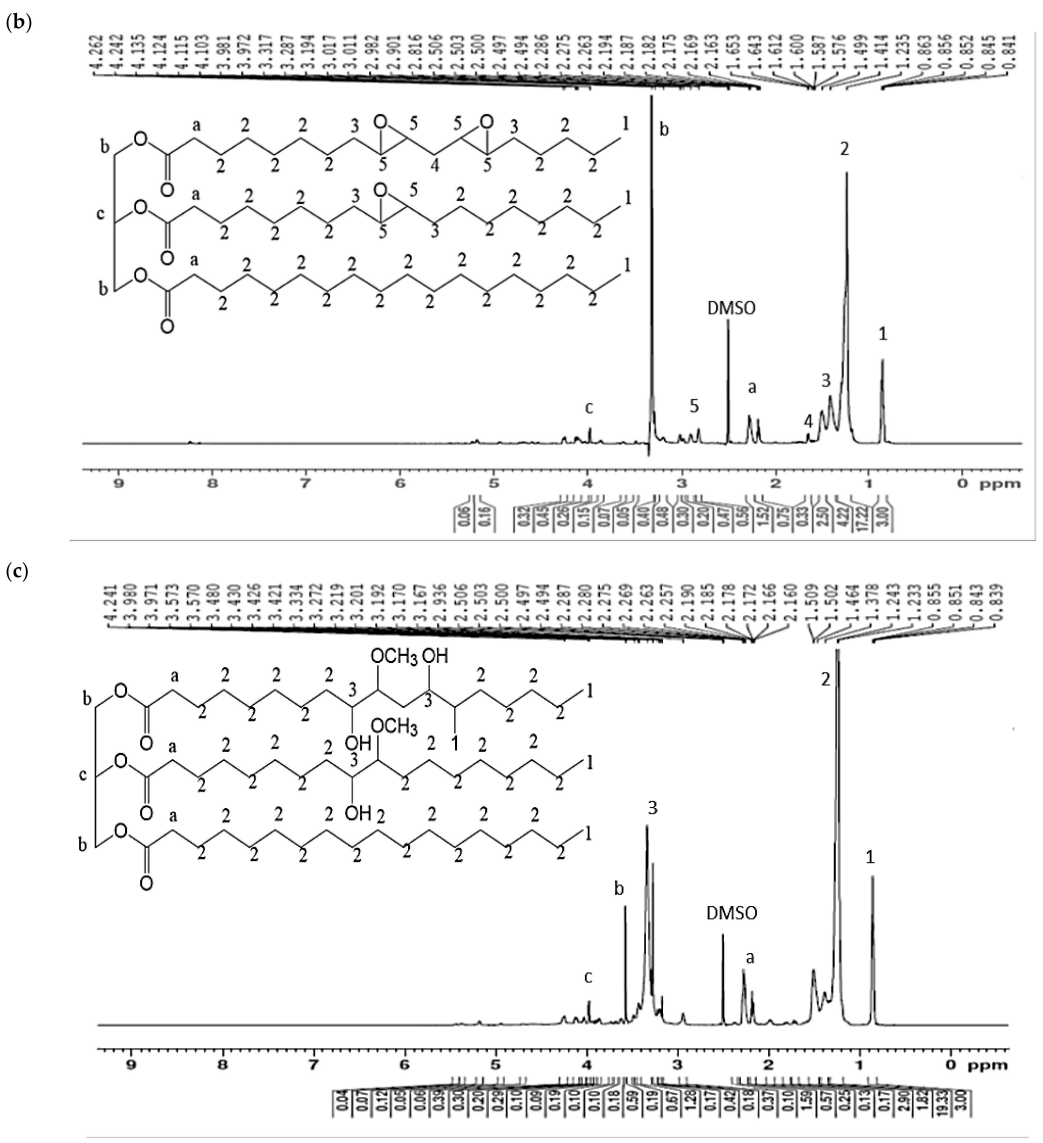
^1^H NMR spectra and the predicted molecular structures of (**a**) JO, (**b**) EJO and (**c**) JOL.

In the previous study by the same authors, the FTIR analysis was conducted at each step of the chemical reaction to produce JPUA-TDI and JPUA-IPDI. However, FTIR only detected the existence of the functional group where both JPUA-TDI and JPUA-IPDI produced a similar trend due to owning a similar functional group [21]. Therefore, the success of the synthesis of JPUA resins was further confirmed with ^1^H NMR analysis. The ^1^H NMR can predict the molecular structure based on hydrogen shifting. The spectra of ^1^H NMR of JO, EJO and JOL are shown in Figure 1. 

In general, JO, EJO, JOL, JPUA-TDI and JPUA-IPDI inherit similar features of the Jatropha oil triglyceride backbone. For example, the signals around 2.2 to 2.3 ppm, 3.5 to 3.7 ppm and 4.00 to 4.30 ppm indicated glycerol moieties of (OCO–C**H**_2_), (R–OCH_2_–CH(OR’)–C**H**_2_OH) and (ROCH_2_–C**H**(OR’)–CH_2_OR), respectively [41]. In the ^1^H NMR spectra, those moieties were identified accordingly as a, b and c. However, during the isocyanation process to produce the JPUA-TDI and JPUA-IPDI, the chemical structures became longer and more complex. Therefore, as mentioned by Abril-Milán and co-workers, the analysis of the proton NMR spectra for PU was conducted on the basis of a certain part of the fatty acid. This involved the existence of active functional groups such as hydroxyl of the fatty acid chain. However, the analysis was relatively valid to give important information for an average chemical structure of the whole PU [42]. In this study, it is noted that some of the glycerol moieties probably did not show any signal during the ^1^H NMR analysis, as it is too small compared to the whole chemical structure of the jatropha oil-based PUA.

In Figure 1a, the main characteristic of JO was shown by the proton of the unsaturated carbon double bond (–**H**C=CH–) that was detected at 5.35 ppm under ^1^H NMR. Apart from that, the signal of the methylene group adjacent to the proton of the carbon double bond (RC**H**_2_CH=CH–) was spotted at 2.02 ppm. Meanwhile, the peak of 2.75 ppm was attributed to the methylene protons between the two carbon double bonds (=CH–C**H**_2_–CH=), which proved the existence of linoleic fatty acid in the triglyceride of JO molecules. This result was comparable with other studies on pure JO [43,44].

In the epoxidation step, the carbon double bond (C=C) in the JO sample was converted into an epoxy (C–O–C) group. In Figure 1b, the signal of the proton double bond (–**H**C=CH–) at 5.35 ppm was diminished. However, there was a trace of an unsaturated part of the carbon double bond in the EJO sample, which is shown by a small peak of 2.8 ppm that possibly belongs to (=CH–C**H**_2_–CH=). Nevertheless, the existence of epoxy features was shown by the presence of the methine of the epoxy group in EJO (–**H**C–O–CH–) at the 2.90 to 3.00 ppm region. Apart from that, the signal at 1.41 to 1.57 ppm was recognised as a methylene proton adjacent to the epoxy group (–HC–O–CH–C**H**_2_–), whereas the chemical shift at 1.65 ppm was attributed to the methylene proton adjacent in between two epoxy groups (HCOCH–C**H**_2_–HCOCH). Similar observations have been revealed by other researchers on palm and jatropha oil-based epoxy resin [38,43]. Moreover, the oxygen oxirane content (OOC) value for EJO was reported as 4.25 ± 0.08% per mole in the previous study, further confirming that most of the carbon double bond was converted into the epoxy group during the epoxidation reaction [21].

Next, the EJO underwent hydroxylation where the ring opening reaction of epoxy occurred to form the hydroxyl group. Based on Figure 1c, the proton NMR spectrum of JOL showed the presence of an epoxy ring residues signal around 2.80 to 3.00 ppm and 1.41 to 1.57 ppm, while traces of the carbon double bond at 5.3 ppm appeared with a very small peak and were almost negligible. Nevertheless, there is still a strong justification to prove the success of the hydroxylation reaction. This was evidenced by the new peak that was detected in the range from 3.27 to 3.83 ppm. According to Abril-Milán et al., and Ling et al., this peak corresponded to the (–C**H**–OH) of the hydroxyl group [42,45]. Furthermore, the hydroxyl value (OHV) for JOL was stated as 149.44 ± 0.23 mg KOH/g in the previous work [21]. This evidence confirmed the formation of the hydroxyl group in the JOL sample. The percentages of the functional groups during in the sample of JO, EJO and JOL are summarised in Table 3.

**Figure 2:**
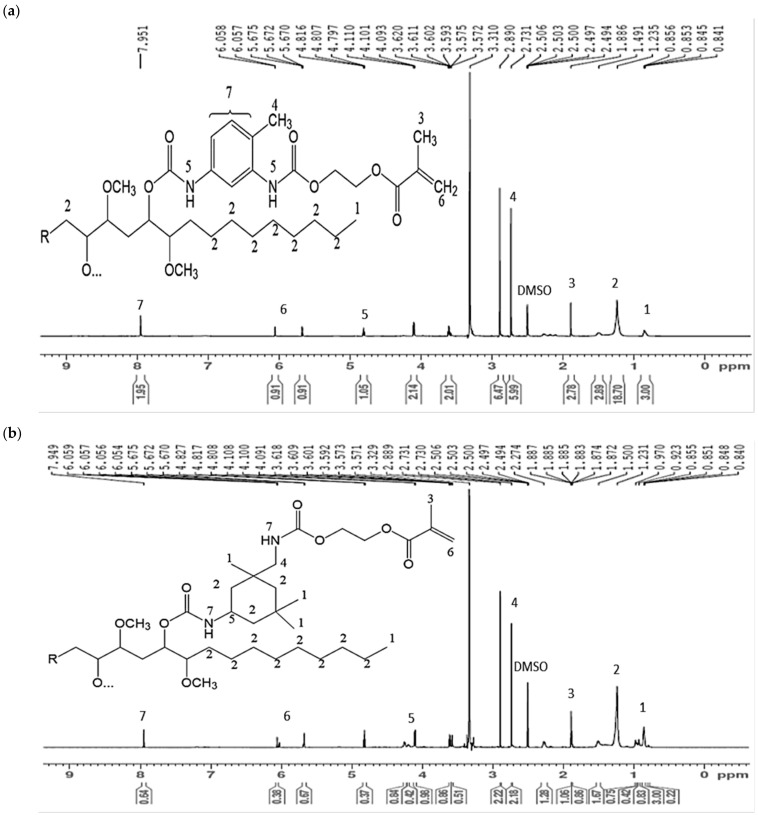
Proton NMR spectra of (**a**) JPUA-TDI and (**b**) JPUA-IPDI and the predicted molecular structure.

For isocyanation, JPUA-TDI was formed by the reaction of JOL with aromatic TDI, while JPUA-IPDI was synthesised from cycloaliphatic IPDI-based diisocyanate. The chemical reactions were terminated with an acrylation reaction with HEMA. The proton NMR spectra of JPUA-TDI and JPUA-IPDI are depicted in Figure 2. Referring to the ^1^H NMR diagram of JPUA-TDI in Figure 2a, the characteristic of the methyl ring protons (Ar–C**H**_3_) of 2,4-TDI was shown at the peak of 2.73 ppm. The presence of the carbamate group (–N**H**–COO) at 4.80 ppm evidenced the reaction between the isocyanate and hydroxyl groups. The formation of a peak of 7.94 ppm explained the possible side reactions between urethane linkages with secondary amine that resulted in the aryl-urea group (Ar–N**H**–COO–). This result has good agreement, as mentioned by Yildiz et al., [46], on structural analysis of polyvinyl butyral (PVB) and TDI-based urethane acrylate oligomer. In addition, the feature given by the acrylate moieties was shown by the chemical shift around 5.68 to 6.06 ppm, which is attributed to the proton carbon double bond, whereas the signal of the methyl group of that attached to the carbon double bond can be seen at a range from 1.79 to 1.83 ppm [43].

Based on the ^1^H NMR diagram of JPUA-IPDI in Figure 2b, the cycloaliphatic proton features and methylene groups (–C**H**_2_–) were mostly attributed by peaks of 1.23 ppm. The peak of 7.94 ppm corresponded to the proton on the carbamate group (–N**H**COO–) in the urethane linkage after reaction between the isocyanate (–NCO) of IPDI with the hydroxyl (–OH) group. Moreover, the proton signal of (R_2_C**H**NCOO–) that attached to the cycloaliphatic group appeared at 4.11 ppm, as reported by Patil et al. [47]. As revealed by Hu et al., the peak of 2.89 ppm was assigned to the methylene group adjacent to the carbamate group (–C**H**_2_NHCOO), which possibly connected to the cycloaliphatic moeities [48]. Similar to JPUA-TDI, the acrylate features of HEMA in JPUA-IPDI showed proton carbon double bonds at the peak from 5.68 to 6.06 ppm. Furthermore, the proton signal of the methyl group of the carbon double bond (C**H**_3_) appeared at 1.90 ppm [46].

### 3.2. Rheological Properties

#### 3.2.1. Effect of Chemical Structure

**Figure 3:**
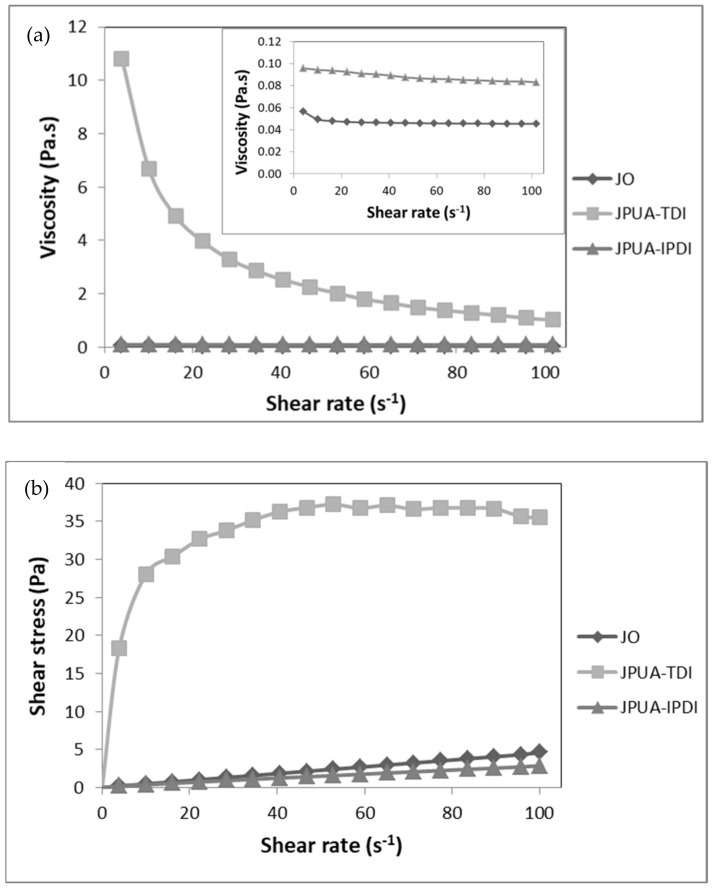
(**a**) Viscosity and (**b**) shear stress relationship with shear rate of JO, JPUA-TDI and JPUA-IPDI.

A rheological study was performed to provide information about how the processing condition such as shear rate and temperature affects the fluid behaviour. Initially, the viscosity and shear stress of the JO, JPUA-TDI and JPUA-IPDI in their virgin form were determined over a shear rate range from 0 to 100 s^−1^ at 25 °C. The rheogram is displayed in Figure 3.

As seen in the graph in the inset of Figure 3a, both JO and JPUA-IPDI showed almost constant viscosity of around 0.05 and 0.09 Pa.s, respectively, within the total range of the applied shear rate. Moreover, a linear relationship between shear stress and shear rate was observed for JO and JPUA-IPDI, as depicted in Figure 3b. These findings reflected the Newtonian fluid behaviour of JO and JPUA-IPDI, as stated by Saalah et al. [49]. In contrast, the nonlinear curve between the shear rate and shear stress in Figure 3b revealed that JPUA-TDI exhibited a non-Newtonian fluid behaviour. In Figure 3a, JPUA-TDI showed pseudoplastic or shear thinning behaviour where a high viscosity was observed at a low shear rate at 25 °C [50].

A liquid can flow due to the ability of the molecules to slide or move between each other. Generally, as the shear rate increased, a higher force would be required to deform the intermolecular force among the molecules in the JO, JPUA-TDI and JPUA-IPDI in order to move in between each other. Thus, the shear stress of JO, JPUA-TDI and JPUA-IPDI increased with the shear rate [51]. Furthermore, the increasing shear rate allowed less resistance to the flow of JO, JPUA-TDI and JPUA-IPDI and, thus, decreased the viscosity value [52].

JPUA-TDI had the highest viscosity followed by JPUA-IPDI and JO. Based on the structure investigation as illustrated in Figure 1, JO in its natural form had the simplest chemical structure compared to JPUA-TDI and JPUA-IPDI as it did not have any chemical modification of the triglycerides chain. Meanwhile, JPUA-TDI and JPUA-IPDI were produced after JOL was reacted with 2,4-TDI and IPDI-based isocyanate, respectively. The chemical modification created a longer polymer chain with higher crosslinking and, thus, produced JPUA-TDI and JPUA-IPDI that were more viscous compared to the JO [12]. This fact is supported with the molecular weight data from the previous study of the same authors. JO has the lowest molecular weight value of 1278 g/mol, followed by JPUA-IPDI (3151 g/mol) and JPUA-TDI (6871 g/mol) [21].

**Figure 4:**
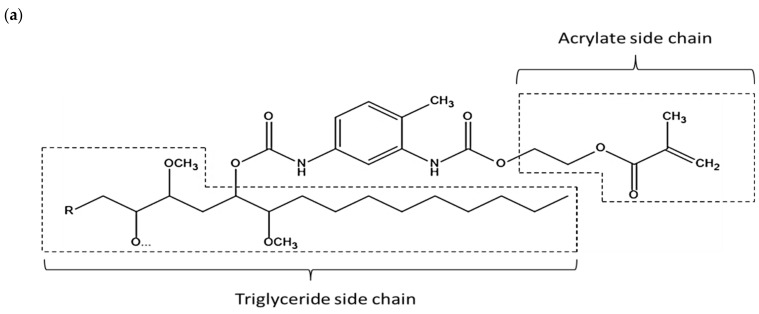

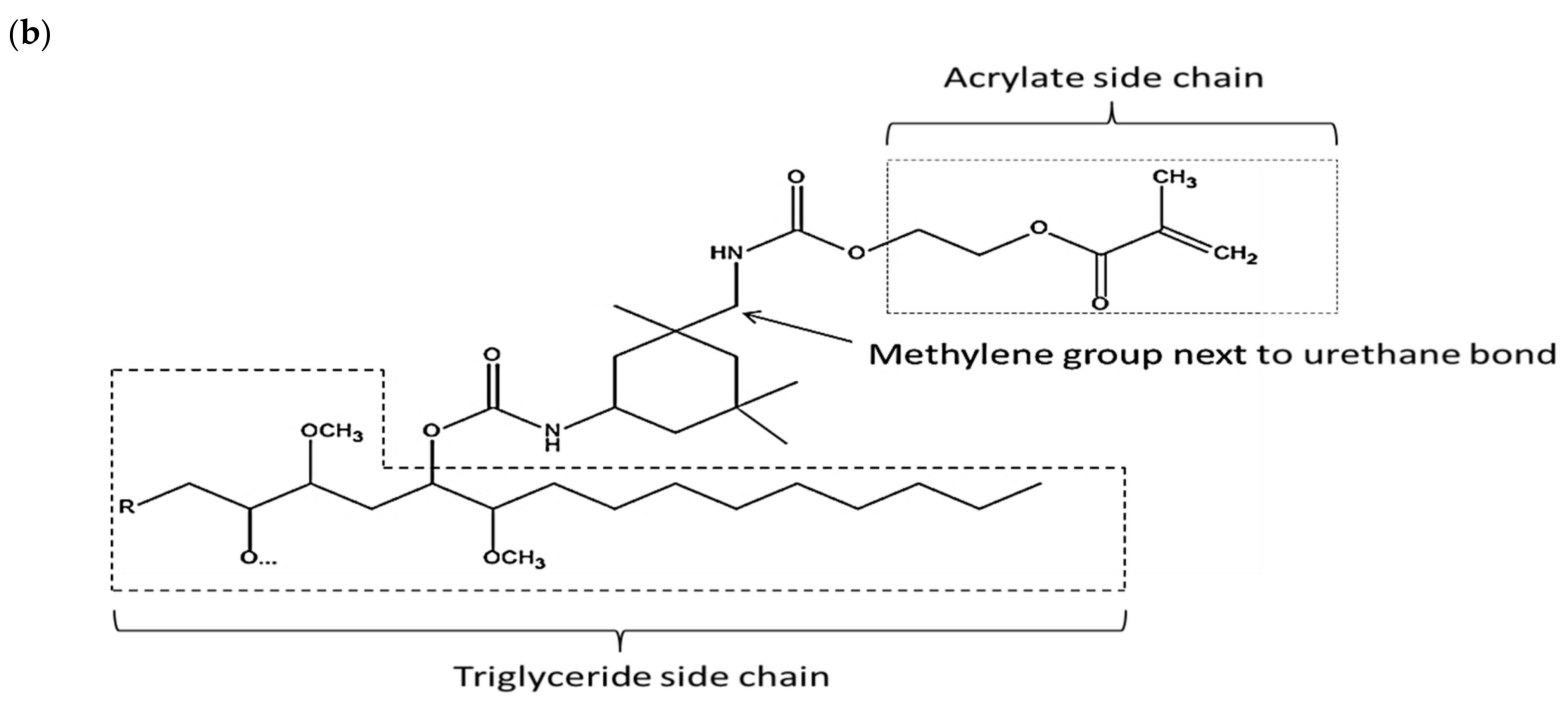
Details of side chain on (**a**) JPUA-TDI and (**b**) JPUA-IPDI structure.

In addition, the viscosity of JPUA-TDI and JPUA-IPDI related to the shape of their molecules is described in Figure 4. JPUA-TDI consisted of a benzene ring with a urethane linkage (-NHCOO-) at the ortho- (1,2) and para- (1,4) position [53]. The close position between the acrylate and triglyceride side chain resulted in a greater entanglement in the JPUA-TDI molecules. 

Meanwhile, the presence of the methylene group (–CH_2_–) in between the cycloaliphatic ring and urethane bond (–NHCOO–) on the acrylate side chain produced more dangling chains on the JPUA-IPDI structure [54]. Therefore, JPUA-TDI needed more shear stress to disentangle and overcome the stronger intermolecular forces to allow the molecules to slide between each other. This explained the higher viscosity of JPUA-TDI compared to JPUA-IPDI [52].

#### 3.2.2. Effect of Temperature

**Figure 5:**
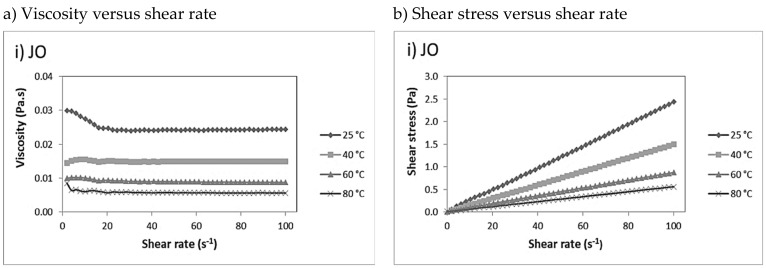

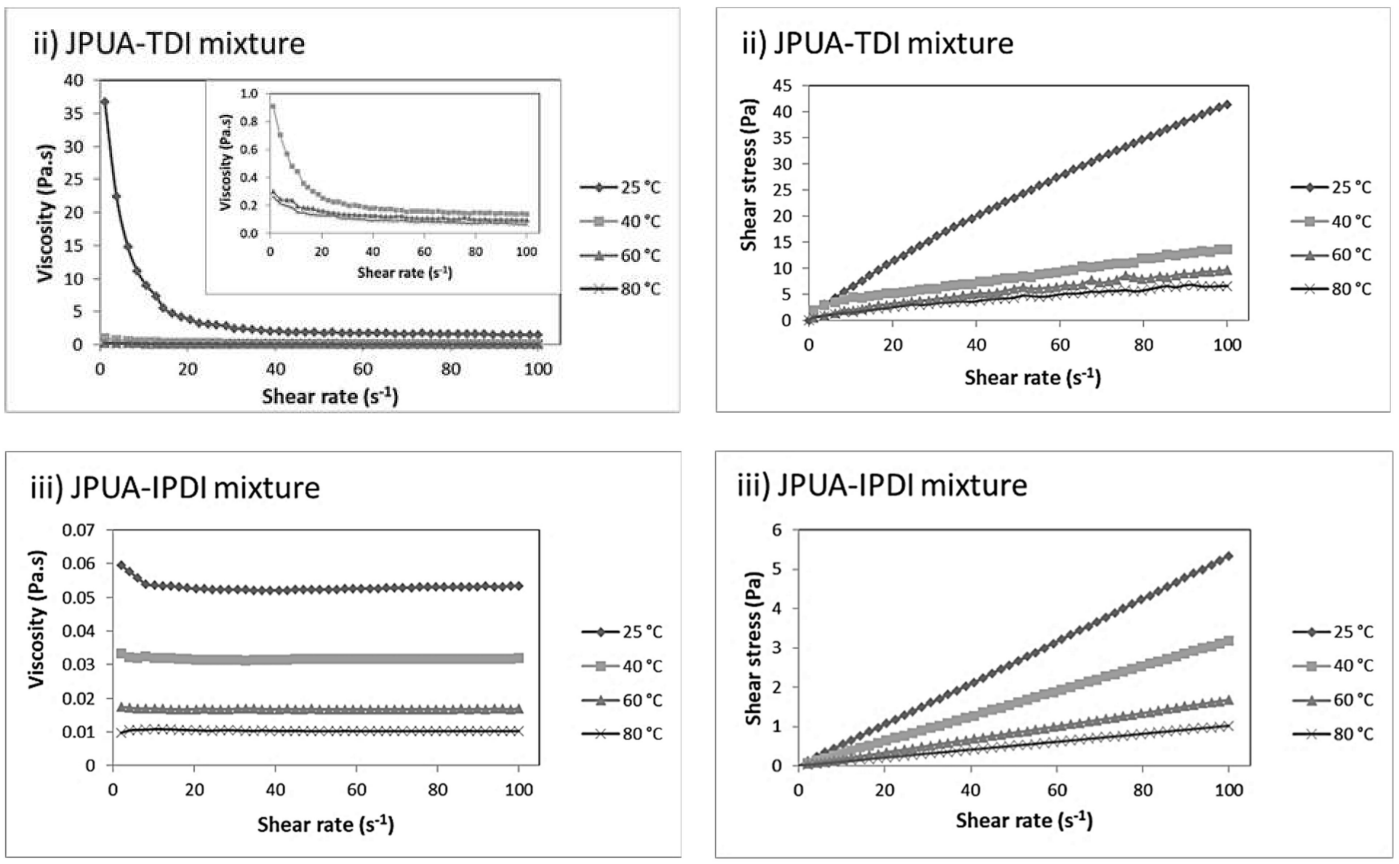
Trends of (**a**) viscosity and (**b**) shear stress at shear rates of 0 to 100 s^−1^ measured at various temperatures. Each set of experiments were conducted on i) JO, ii) JPUA-TDI mixture and iii) JPUA-IPDI mixture respectively.

The viscosity and shear stress of the JO, JPUA-TDI mixture and JPUA-IPDI mixture were examined at 25 °C, 40 °C, 60 °C and 80 °C, as shown in Figure 5. Those temperatures were selected to determine the flow behaviour of the samples at both room temperature and during processing parameters [50].

For both the JPUA-TDI mixture and JPUA-IPDI mixture, TMPTA was added as a reactive diluent in the uncured coating formulation. The presence of TMPTA effectively reduced the viscosity of both types of coating formulation, especially for the JPUA-TDI mixture [55,56]. Generally, the viscosity of the JO, JPUA-TDI mixture and JPUA-IPDI mixture reduced as the temperature increased. This was due to the increasing temperature which led to the rise in the average kinetic energy in the molecules in the uncured coating formulation solution. Therefore, the intermolecular forces in the polymer became weaker and the molecules were allowed to flow more freely [51]. As a result, the viscosity showed reducing trends with the increase in temperature. A similar observation was reported by Diamante and co-workers for walnut oil and rice bran oil [57] and also by a team led by Zahir regarding corn oil and mustard oil [58] at increasing temperature. As less force was required to overcome the intermolecular force, the shear stress also showed a reducing trend during elevated temperatures [52].

**Figure 6:**
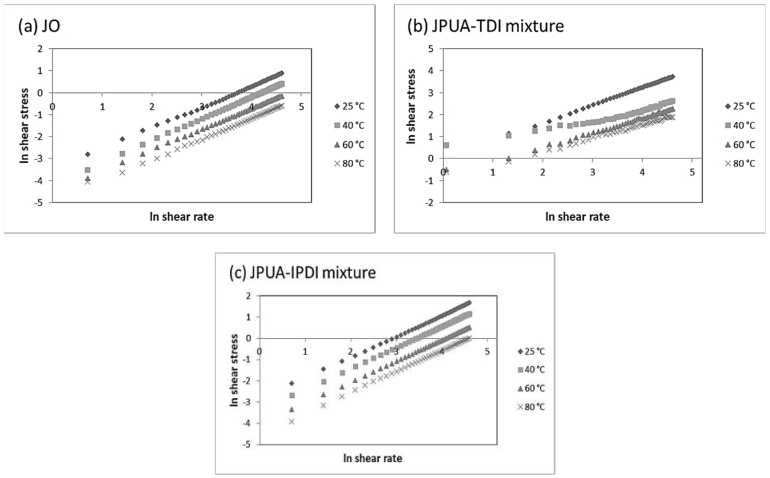
Graph of ln shear stress versus ln shear rate of (**a**) JO, (**b**) JPUA-TDI mixture and (**c**) JPUA-IPDI mixture.

In order to determine the k and n value, the graph of ln shear stress versus ln shear rate was plotted for the JO, JPUA-TDI mixture and JPUA-IPDI mixture, as illustrated in Figure 6. The k and n values were determined based on the y-intercept and slope of the graph, respectively. The related information is presented in Table 4.

Theoretically, the k value reflects the fluid viscosity, and the n value gives information concerning the fluid behaviour, as explained below [50]:

n > 1 = shear thickening

n = 1 = Newtonian fluid

n < 1 = shear thinning

The JO and JPUA-IPDI mixture exhibited Newtonian characteristics at 25 °C, 40 °C, 60 °C and 80 °C, where the n value was near to 1, as listed in Table 3. In contrast, the JPUA-TDI mixture demonstrated a shear thickening fluid behaviour at 25 °C with an n value more than 1. At 40 °C, 60 °C and 80 °C, the JPUA-TDI mixture exhibited a shear thinning behaviour in which the n value was less than 1. From this, it was concluded that the temperature had a significant effect on the fluid behaviour, especially for the JPUA-TDI mixture solution. This findings are in good agreement with a study revealed by Parcheta and Datta in determining the fluid behaviour of bio-based polyols [50].

#### 3.2.3. Development of Master Curve Graph

**Figure 7:**
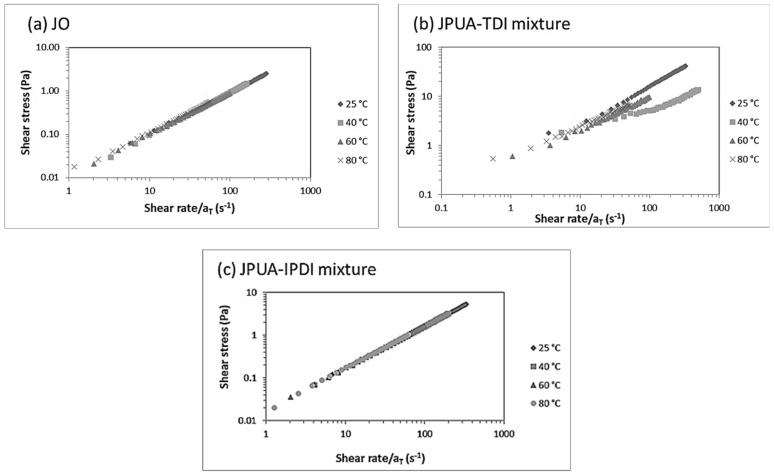
Shear rate-based master curve graphs of (**a**) JO, (**b**) JPUA-TDI and (**c**) JPUA-IPDI at 25 °C, 40 °C, 60 °C and 80 °C.

Details of the calculation to determine the shear rates (with σ = 0.5 Pa* or 5.0 Pa**) and shift factor values are tabulated in Table 5, while the master curve graph of the JO, JPUA-TDI mixture and JPUA-IPDI mixture is presented in Figure 7.

Based on Figure 7, the JO and JPUA-IPDI mixture have established horizontal shifting and produced a single line of data overlapping at shear rates of 0 to 100 s^−1^. This indicated that the information regarding the JO and JPUA-IPDI mixture fluid behaviour at different temperatures was successfully combined in a single data sheet for feasibility studies and for future reference. However, as the JPUA-TDI mixture was a non-Newtonian fluid with shear thinning and shear thickening, the data obeyed a perfect single line only until the shear rate of 70 s^−1^. For this reason, it was challenging to utilise the non-Newtonian fluid for the encapsulation process as the trend was sometimes unpredictable. The JPUA-TDI mixture was discarded due to the possibility of considerable difficulty in the processing, as reported by other researchers, such as agglomeration issues, sticky microcapsules [59] and low core content [31]. Therefore, only the JO and JPUA-IPDI mixtures were chosen for the encapsulation process in the next experiment. 

## 4. Conclusions

Success of the chemical reaction in producing JPUA-TDI and JPUA-IPDI was confirmed via ^1^H NMR analysis via the presence of urethane linkage features. The chemical structure was predicted based on proton shifting. The chemical structure of the JO, JPUA-TDI and JPUA-IPDI affected their rheological behaviour. The viscosity of the resin was determined in the manner of JPUA-TDI > JPUA-IPDI > JO related to their structural conformation. For the JPUA-TDI mixture and JPUA-IPDI mixture, the addition of a TMPTA monomer effectively reduced the fluid viscosity. At 25 °C, 40 °C, 60 °C and 80 °C, the JO and JPUA-IPDI mixture exhibited Newtonian behaviour and showed single overlapping data when presented in the master curve graph over the whole shear rate range. The JPUA-TDI mixture showed a shear thickening fluid at 25 °C and shear thinning behaviour at 40 °C, 60 °C and 80 °C. Apart from that, the JPUA-TDI mixture revealed an unstable fluid behaviour as it only conformed in a single line until a shear rate of 70 s^−1^ in the master curve graph. In order to minimise problems such as agglomeration and sticky microcapsules, the JPUA-TDI mixture was discarded and only the JO and JPUA-IPDI mixture were chosen to proceed for the encapsulation experiment, which targeted self-healing coating applications using air or the UV mode of repairing.

## Figures and Tables

**Table 1:** Fatty acid components in jatropha oil.

Fatty Acid	Amount (%)
Palmitic	12.8
Stearic	7.3
Oleic	41.3
Linoleic	34.4
Unsaturated	2.7

**Table 2:** Components of JO, JPUA-TDI and JPUA-IPDI-based mixture resin.

Code	JO (%)	JPUA-TDI (%)	JPUA-IPDI (%)	TMPTA (%)	Benzophenone (%)
JO	100	-	-	-	-
JPUA-TDI	-	100	-	-	-
JPUA-IPDI	-	-	100	-	-
JPUA-TDI mixture	-	65	-	35	4
JPUA-IPDI mixture	-	-	65	35	4

**Table 3:** Percentages of carbon double bond, epoxy and hydroxyl functional groups in JO, EJO and JOL sample.

Sample	Functional Group per One Mole of Triglyceride (%)		
	Carbon Double Bond	Epoxy	Hydroxyl
JO	20.16	-	-
EJO	2.06	22.56	-
JOL	0.30	5.27	12.47

**Table 4:** Power law fluid properties of JO, JPUA-TDI mixture and JPUA-IPDI mixture at four different temperatures.

Sample	Temperature (°C)	Linear Equation	k	n
JO	25	y = 0.9469x − 3.5035	0.030	0.947
	40	y = 0.9971x − 4.1949	0.015	0.997
	60	y = 0.953x − 4.5343	0.011	0.953
	80	y = 0.935x − 4.9147	0.007	0.935
JPUA-TDI mixture	25	y = 1.0997x − 1.075	0.341	1.100
	40	y = 0.3022x + 0.9576	2.605	0.302
	60	y = 0.3301x + 0.4313	1.539	0.330
	80	y = 0.4672x − 0.3607	0.697	0.467
JPUA-IPDI mixture	25	y = 0.9825x − 2.8722	0.057	0.983
	40	y = 0.9945x − 3.4319	0.032	0.994
	60	y = 0.9941x − 4.0659	0.017	0.994
	80	y = 0.9912x − 4.5459	0.011	0.991

**Table 5:** Shear rates and shift factor values with reference to calculated **˙γ** at 60 °C.

Sample	Temperature (°C)	˙γ at 0.5 Pa*/5 Pa** (s^−1^)	Calculated ˙γ (s^−1^)	a_T_ (˙γ/˙γ_60_)
JO *	25	20.4	19.51	1.293
	40	34.7	33.69	1.197
	60	57.1	54.87	0.999
	80	89.8	96.11	0.877
JPUA-TDI mixture **	25	8.5	11.49	0.002
	40	18.5	8.65	0.141
	60	40.8	35.54	1.000
	80	61.2	67.99	0.165
JPUA-IPDI mixture *	25	10.2	9.11	1.396
	40	16.3	15.89	1.190
	60	30.6	30.02	1.000
	80	51.0	47.06	0.891

*shear rate at 0.5 Pa, ** shear rate at 5.0 Pa.

## Data Availability

The data presented in this study are available on request from the corresponding author.
